# The metabolic effects of adding exenatide to basal insulin therapy when targeting remission in early type 2 diabetes in a randomized clinical trial

**DOI:** 10.1038/s41467-022-33867-9

**Published:** 2022-10-16

**Authors:** Ravi Retnakaran, Chang Ye, Alexandra Emery, Caroline K. Kramer, Bernard Zinman

**Affiliations:** 1 Leadership Sinai Centre for Diabetes, Mount Sinai Hospital, Toronto, Canada; 2 Division of Endocrinology, University of Toronto, Toronto, Canada; 3 Lunenfeld-Tanenbaum Research Institute, Mount Sinai Hospital, Toronto, Canada

**Keywords:** Type 2 diabetes, Medical research, Clinical trials

## Abstract

Combining a glucagon-like peptide-1 receptor agonist (GLP1-RA) with basal insulin is an emerging option when initiating injectable therapy in longstanding type 2 diabetes (T2DM). Recognizing that short-term insulin therapy can improve beta-cell function and induce glycemic remission in early T2DM, we hypothesized that adding the short-acting GLP1-RA exenatide to basal insulin in early T2DM may enhance the achievability of these outcomes. In this completed, 20-week, open-label, parallel-arm trial at an academic hospital, 103 individuals aged 30–80 years with <7 years duration of T2DM were randomized (by computer-generated sequence) to 8-weeks treatment with (i) insulin glargine (Glar; *n* = 33), (ii) glargine + thrice-daily lispro (Glar/Lispro; *n* = 35), or (iii) glargine + twice-daily exenatide (Glar/Exenatide; *n* = 35), followed by 12-weeks washout. The analyzed population of 102 participants (median 3.5 years of T2DM, A1c 6.6% ±0.7%) consisted of 33 on Glar, 35 on Glar/Lispro and 34 on Glar/Exenatide. Oral glucose tolerance tests at baseline, 4-weeks, 8-weeks and 20-weeks enabled assessment of beta-cell function (Insulin Secretion-Sensitivity Index-2 (ISSI-2)) and glycemic control. Mean ISSI-2 over the 8-week intervention (primary outcome) did not differ across the groups (Glar/Exenatide 237 ± 11; Glar/Lispro 208 ± 11; Glar 223 ± 11; *p* = 0.19). Baseline-adjusted A1c at 8-weeks (secondary outcome) was lowest in Glar/Exenatide followed by Glar/Lispro and Glar (mean 5.9% vs 6.0% vs 6.2%; *p* = 0.0007). After 12-weeks washout, however, neither baseline-adjusted A1c nor baseline-adjusted ISSI-2 (secondary outcomes) differed between the groups, nor did (additional outcome) rates of remission (Glar/Exenatide 26.7%, Glar/Lispro 43.8%, Glar 32.1%; *p* = 0.35). There were no severe hypoglycemia episodes. In conclusion, adding exenatide to basal insulin in early T2DM does not further enhance underlying beta-cell function or the capacity to achieve diabetes remission, despite yielding on-treatment glycemic benefit.

## Abstract (web summary)

Combining a GLP-1 receptor agonist, to increase endogenous beta cell function, with exogenous insulin is a possible therapeutic option for the treatment of type 2 diabetes. Here, the authors show adding exenatide to basal insulin in early type 2 diabetes does not further enhance underlying pancreatic beta-cell function or the capacity to achieve diabetes remission, but may yield on-treatment glycemic benefits.

## Introduction

Combination therapy consisting of a glucagon-like peptide-1 receptor agonist (GLP1-RA) and basal insulin has emerged as an effective therapeutic option in the management of longstanding type 2 diabetes (T2DM)^1, 2^. In clinical trials and meta-analyses thereof^3–7^, the complementary effects of GLP1-RA (regulating post-prandial glycemia) and basal insulin (regulating post-absorptive and fasting glycemia) have translated clinically into this combination yielding an ideal therapeutic trifecta – namely, robust glucose-lowering coupled with mitigation of the typical insulin-associated risks of hypoglycemia and weight gain^5^. These enticing features have led to the suggestion that combined treatment with basal insulin and GLP1-RA warrants consideration when first initiating injectable therapy in patients with longstanding T2DM and sub-optimal glycemic control on oral medications^8^. Moreover, these data raise the intriguing question of whether initiating this combination therapy early in the course of T2DM may offer enhanced metabolic benefit.

When administered in early T2DM, a short course of insulin therapy for 2–4 weeks can improve beta-cell dysfunction and even induce glycemic remission^9–11^. While these outcomes have most commonly been achieved with intensive insulin therapy (either multiple daily injections or continuous subcutaneous insulin infusion), basal insulin alone can also yield such benefits^12^. Moreover, the Outcome Reduction with an Initial Glargine Intervention (ORIGIN) Trial found that, in patients with pre-diabetes, treatment with insulin glargine significantly reduced the risk of developing T2DM, with partial protection still evident ~3 months after stopping the treatment^13^. However, in practice, the implementation of early insulin therapy can be limited by patient and provider fear of the risks of hypoglycemia and weight gain^14^. Furthermore, the degree of beta-cell functional recovery in response to this intervention can be heterogeneous and the induced remission of diabetes is not permanent, ultimately waning over time^10, 15^.

In this context, we hypothesized that combining basal insulin with a GLP1-RA in early T2DM may provide a strategy that could enhance the achievability of the desired metabolic outcomes of improved beta-cell function and diabetes remission, while limiting the risks of hypoglycemia and weight gain. Since it is believed that short-acting GLP1-RAs act preferentially on post-prandial glycemia through their inhibitory effect on gastric emptying (in contrast to long-acting formulations that have more pronounced effects on fasting and post-absorptive glycemia)^2, 16–18^, we reasoned that combining the short-acting GLP1-RA exenatide with basal insulin may provide optimal complementarity in metabolic coverage. Indeed, recognizing that the postprandial glucose-lowering effect of exenatide correlates with the magnitude of its inhibitory effect on the rate of gastric emptying^17^, this short-acting GLP1-RA should reduce the insulin secretory demands placed on the beta-cells, thereby inducing a degree of beta-cell rest (akin to the effect of bolus insulin in short-term intensive insulin therapy)^10, 15^.

In this clinical trial, we sought to determine whether adding exenatide to basal insulin therapy in early T2DM can enhance the achievability of beta-cell functional recovery and diabetes remission, as compared to basal/bolus therapy and basal insulin alone. We demonstrate that the addition of exenatide to basal insulin does not further enhance underlying beta-cell function or the capacity to achieve diabetes remission in early T2DM but does yield on-treatment glycemic and weight benefits.

## Results

**Fig. 1:**
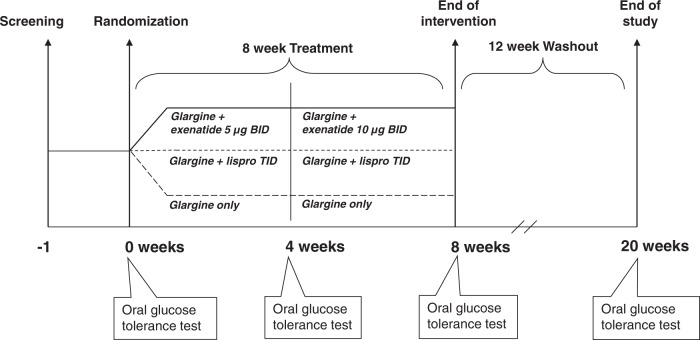
Flow diagram showing study design.

The PREserVing Beta-cell Function in Type 2 Diabetes with Exenatide And InsuLin **(**PREVAIL) Trial was an open-label, parallel-arm, randomized controlled trial that was designed to evaluate the metabolic effects of three insulin-based therapies (glargine alone, glargine + lispro, glargine + exenatide) early in the course of T2DM. In this 20-week trial, participants were randomized (1:1:1) to one of these interventions for 8-weeks, followed by a subsequent 12-week washout off any anti-diabetic medication. As shown in Fig. 1, participants underwent assessment of beta-cell function, insulin sensitivity and glucose homeostasis by oral glucose tolerance test (OGTT) at baseline, 4-weeks, 8-weeks, and 20-weeks (i.e. after 12-week washout).

**Fig. 2:**
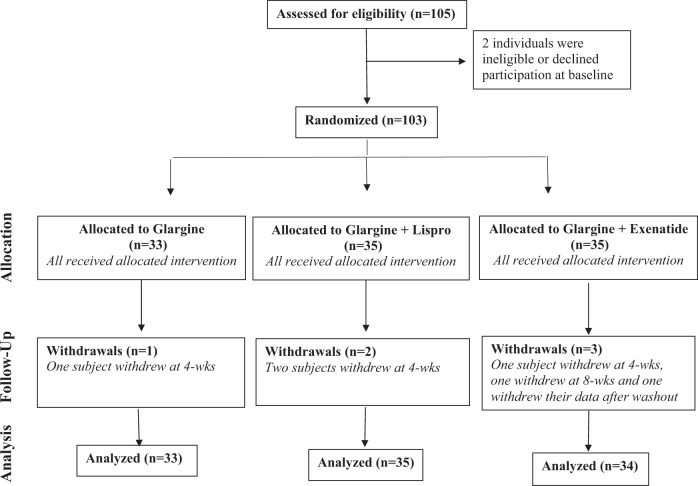
CONSORT (Consolidated Standards of Reporting Trials) profile showing flow of trial participants.

Figure 2 shows the trial profile. We recruited 103 participants, one of whom withdrew their data after completing the study. Participants were recruited from the practices of family physicians (either by screening charts or physician referral) and in response to advertising of the study. The analyzed study population consisted of 102 participants with T2DM of median 3.5 years duration (interquartile range 1.8–5.5 years), baseline A1c 6.6% ±0.7% and BMI 31.9 ± 7.5 kg/m^2^. These participants were randomized to one of three interventions: (I) insulin glargine alone (Glar; *n* = 33), (ii) glargine + pre-prandial lispro (Glar/Lispro; *n* = 35), or (iii) glargine + twice-daily exenatide (Glar/Exenatide, *n* = 34). Baseline characteristics of the groups showed no significant differences in clinical or metabolic measures (Table 1). The initial daily dose of glargine was similar across the three groups: Glar 0.11 ± 0.01 units/kg; Glar/Lispro 0.11 ± 0.01 units/kg; Glar/Exenatide 0.12 ± 0.01 (*p* = 0.82). At 8-weeks, the final daily doses of glargine were as follows: Glar 0.42 ± 0.23 units/kg; Glar/Lispro 0.36 ± 0.19 units/kg; Glar/Exenatide 0.33 ± 0.24 units/kg (*p* = 0.24). Six participants withdrew during the trial (1 Glar, 2 Glar/Lispro, and 3 Glar/Exenatide). Adherence to the protocol was high and was supported by the observed improvements in glycemic control during the study on SMBG and laboratory measurements. The glucose and insulin responses on the OGTT at baseline, 8-weeks and washout are shown in Supplementary Figs. 1 and 2, respectively. Of note, all three interventions induced significant reduction in A1c at 8-weeks, with mean changes from baseline as follows: Glar: −0.3% (95% CI −0.5, −0.1), *p* = 0.002; Glar/Lispro: −0.5% (−0.7, −0.3), *p* < 0.0001; Glar/Exenatide: −0.7% (−0.8, −0.5); p < 0.0001). There were early washout visits in 19 participants (7 Glar, 7 Glar/Lispro, 5 Glar/Exenatide).

**Table 1:** Baseline characteristics of the three groups: (I) Glargine, (II) Glargine + Lispro, and (III) Glargine + Exenatide

	(Group I)	(Group II)	(Group III)	Overall *P*
	Glargine (*n * = 33)	Glargine + Lispro (*n* = 35)	Glargine + Exenatide (*n* = 34)	
Age (years)	58 ± 10	59 ± 9	56 ± 10	0.40
Sex (% male)	19 (57.6)	16 (45.7)	23 (67.7)	0.18
Ethnicity:	–	–	–	0.65
White (%)	23 (69.7)	21 (60.0)	20 (58.8)	–
South Asian (%)	2 (6.1)	4 (11.4)	6 (17.7)	–
Other (%)	8 (24.2)	10 (28.6)	8 (23.5)	–
Duration of diabetes (years)	3.0 (1.9–5.5)	4.0 (1.9–5.8)	3.9 (1.5–5.3)	0.87
Retinopathy (%)	0 (0)	0 (0)	0 (0)	–
Proteinuria (%)	3 (9.1)	3 (8.6)	3 (8.8)	0.99
Neuropathy (%)	4 (12.1)	3 (8.6)	3 (8.8)	0.84
DM medications before study:	–	–	–	0.72
Lifestyle only (%)	5 (15.2)	3 (8.6)	8 (23.5)	–
Metformin (%)	20 (60.6)	22 (62.9)	19 (55.9)	–
DPP-4 inhibitor (%)	1 (3.0)	0 (0)	0 (0)	–
SGLT-2 inhibitor (%)	0 (0)	0 (0)	1 (2.9)	–
Sulfonylurea (%)	0 (0)	2 (5.7)	0 (0)	–
Metformin + DPP-4 inhibitor (%)	6 (18.2)	6 (17.1)	5 (14.7)	–
Metformin + SGLT-2 inhibitor (%)	1 (3.0)	1 (2.9)	1 (2.9)	–
Metformin + Sulfonylurea (%)	0 (0)	1 (2.9)	0 (0)	–
Weight (kg)	93.7 ± 18.1	88.9 ± 18.2	93.0 ± 23.3	0.56
Body mass index (kg/m^2^)	32.3 ± 6.6	32.9 ± 8.4	31.7 ± 7.6	0.94
Waist circumference (cm)	108.4 ± 14.5	104.7 ± 15.0	107.0 ± 16.9	0.62
Glycemia:	–	–	–	–
A1c (%)	6.6 ± 0.7	6.5 ± 0.8	6.6 ± 0.6	0.83
A1c (mmol/mol)	48.8 ± 8.1	47.6 ± 9.0	48.3 ± 6.6	0.83
Fasting glucose (mmol/l)	7.1 ± 1.6	6.9 ± 1.5	7.0 ± 1.2	0.91
Insulin sensitivity/resistance:	–	–	–	–
Matsuda index	2.3 (1.8–3.1)	2.3 (1.2–3.8)	2.6 (1.6–4.1)	0.99
HOMA-IR	3.9 (3.3–6.7)	4.2 (2.6–9.0)	3.5 (2.3–7.3)	0.95
Beta-cell function:				
ISSI-2	195 (103–268)	171 (114–244)	192 (118–237)	0.89
Insulinogenic index/HOMA-IR	1.6 (0.9–2.4)	1.4 (0.7–2.7)	3.5 (2.3–7.3)	0.96
ΔCpeptide_0-120_/Δgluc_0-120_ × Matsuda	822 (313–1433)	530 (388–1421)	672 (333–1129)	0.69
ΔISR_0-120_/Δgluc_0-120_ × Matsuda	2.7 (1.1–5.0)	2.0 (1.3–6.1)	2.7 (1.2–4.8)	0.80

Continuous variables are presented as mean followed by standard deviation in parentheses (if normal distribution) or median followed by interquartile range (if skewed distribution). Categorical variables are presented as proportions.

The primary, secondary and additional outcomes are shown in Table 2. The primary outcome of mean ISSI-2 over the 8-week intervention did not differ across the groups (Glar/Exenatide 237 ± 11; Glar/Lispro 208 ± 11; Glar 223 ± 11; *p* = 0.19). The secondary outcome of baseline-adjusted A1c at 8-weeks was lowest in Glar/Exenatide followed by Glar/Lispro and Glar (mean 5.9% vs 6.0% vs 6.2%; *p* = 0.0007). Additional outcomes at 8-weeks revealed that baseline-adjusted weight and BMI were lowest in Glar/Exenatide (both *p* = 0.0001), coupled with lower baseline-adjusted 2-hour blood glucose on the OGTT (*p* = 0.045). There were otherwise no significant differences between the groups in three additional measures of baseline-adjusted beta-cell function at 8-weeks (insulinogenic index/HOMA-IR; ΔCpeptide_0-120_/Δgluc_0-120_ × Matsuda index; ΔISR_0-120_/Δgluc_0-120_ × Matsuda index), or in baseline-adjusted insulin sensitivity (Matsuda index) or insulin resistance (HOMA-IR). Since all participants were negative for anti-glutamic acid decarboxylase antibodies except for one individual in which the assay was indeterminate, we ran sensitivity analyses in which the latter individual was excluded and confirmed that findings for all outcomes were unchanged.

**Table 2:** Primary, secondary and additional outcomes at 8-weeks and 20-weeks

	Glargine	Glargine + Lispro	Glargine + Exenatide	Overall *P*
	(*n* = 33)	(*n* = 35)	(*n* = 34)	
(I) Primary outcome:				
Mean ISSI-2 over 8-week intervention	223 ± 11	208 ± 11	237 ± 11	0.19
(II) Secondary outcomes:				
Baseline-adjusted A1c at 8-weeks (%)	6.2 ± 0.07	6.0 ± 0.06	5.9 ± 0.07	0.0007
Baseline-adjusted ISSI-2 at 20-weeks	148.2 ± 12.0	148.6 ± 11.9	171.6 ± 14.4	0.36
Baseline-adjusted A1c at 20-weeks (%)	6.7 ± 0.1	6.6 ± 0.09	6.6 ± 0.1	0.69
(III) Additional outcomes at 8-weeks:				
Additional beta-cell measures:				
Baseline-adjusted insulinogenic index/HOMA-IR	1.6 ± 0.2	1.6 ± 0.2	1.7 ± 0.2	0.92
Baseline-adjusted ΔCpep_0-120_/Δgluc_0-120_ × Matsuda	1117 ± 178	930 ± 143	1288 ± 204	0.34
Baseline-adjusted ΔISR_0-120_/Δgluc_0-120_ × Matsuda	3.8 ± 0.7	3.5 ± 0.6	4.6 ± 0.8	0.50
Insulin sensitivity/resistance:				
Baseline-adjusted Matsuda index	3.1 ± 0.4	2.2 ± 0.2	2.4 ± 0.3	0.08
Baseline-adjusted HOMA-IR	3.2 ± 0.4	3.6 ± 0.4	3.5 ± 0.4	0.74
Additional glycemic measures:				
Baseline-adjusted fasting glucose (mmol/l)	6.0 ± 0.2	6.4 ± 0.2	5.8 ± 0.2	0.16
Baseline-adjusted 2-hr glucose (mmol/l)	13.1 ± 0.5	13.7 ± 0.5	12.1 ± 0.5	0.045
Anthropometric measures:				
Baseline-adjusted weight (kg)	93.5 ± 0.4	92.7 ± 0.4	90.9 ± 0.4	0.0001
Baseline-adjusted BMI (kg/m^2^)	32.5 ± 0.2	32.3 ± 0.1	31.6 ± 0.1	0.0001
Baseline-adjusted waist circumference (cm)	106.6 ± 0.7	107.5 ± 0.6	105.6 ± 0.6	0.11
(IV) Additional outcomes at 20-weeks:				
Additional beta-cell measures:				
Baseline-adjusted insulinogenic index/HOMA-IR	1.1 ± 0.2	1.0 ± 0.2	1.2 ± 0.2	0.85
Baseline-adjusted ΔCpep_0-120_/Δgluc_0-120_ × Matsuda	666 ± 133	700 ± 135	617 ± 127	0.90
Baseline-adjusted ΔISR_0-120_/Δgluc_0-120_ × Matsuda	2.8 ± 0.6	3.0 ± 0.5	2.8 ± 0.5	0.91
Insulin sensitivity/resistance:				
Baseline-adjusted Matsuda index	2.1 ± 0.2	1.8 ± 0.2	2.2 ± 0.2	0.43
Baseline-adjusted HOMA-IR	5.4 ± 0.6	5.0 ± 0.5	4.5 ± 0.5	0.48
Additional glycemic measures:				
Baseline-adjusted fasting glucose (mmol/l)	7.6 ± 0.2	7.6 ± 0.2	7.0 ± 0.2	0.14
Baseline-adjusted 2-hr glucose (mmol/l)	14.9 ± 0.6	14.2 ± 0.6	14.6 ± 0.6	0.63
Anthropometric measures:				
Baseline-adjusted weight (kg)	92.3 ± 0.6	91.8 ± 0.5	90.8 ± 0.5	0.15
Baseline-adjusted BMI (kg/m^2^)	32.1 ± 0.2	31.9 ± 0.2	31.6 ± 0.2	0.13
Baseline-adjusted waist circumference (cm)	105.6 ± 0.8	106.7 ± 0.8	105.0 ± 0.8	0.27

Data are presented as mean ± standard error.

After 12-weeks washout, the additional secondary outcomes of baseline-adjusted ISSI-2 at 20-weeks and baseline-adjusted A1c at 20-weeks showed no significant differences between the groups (*p* = 0.36 and *p* = 0.69, respectively). There were also no significant differences in the additional baseline-adjusted measures of beta-cell function, insulin sensitivity/resistance, glycemia or anthropometry. Thus, the beneficial effects of Glar/Exenatide on glycemic control and weight that were observed after 8-weeks of treatment did not persist in the 3-months that followed after stopping the therapy.

**Fig. 3:**
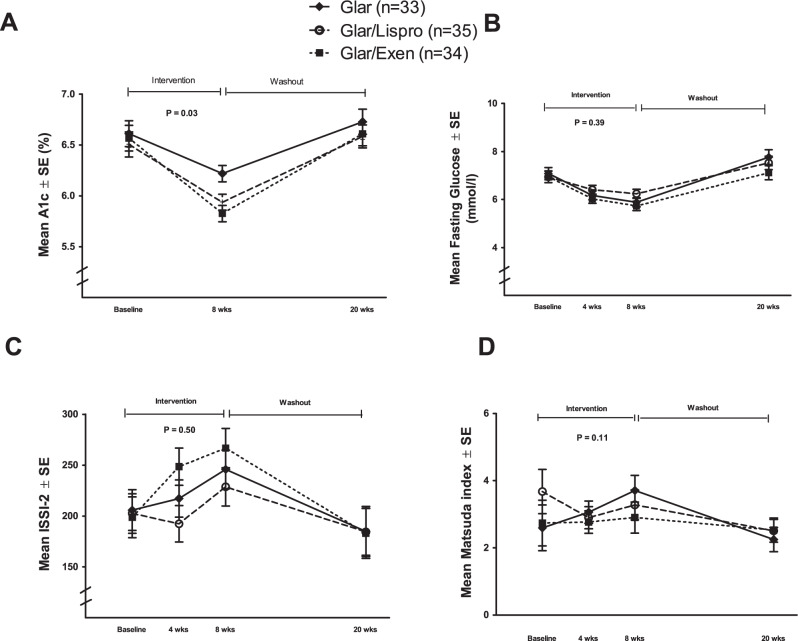
Changes over time in metabolic variables. **A** A1c, **B** fasting glucose, **C** Insulin Secretion-Sensitivity Index-2 (ISSI-2), and **D** Matsuda index. Data are presented as mean values ± standard error. Generalized estimating equation (GEE) model was used to test time-treatment interaction effect during the 8-week intervention. Two-tailed *P*-values < 0.05 were considered statistically significant (multiple comparisons not considered). Number of participants: Glar *n* = 33; Glar/Lispro *n* = 35; Glar/Exen *n* = 34.

**Fig. 4:**
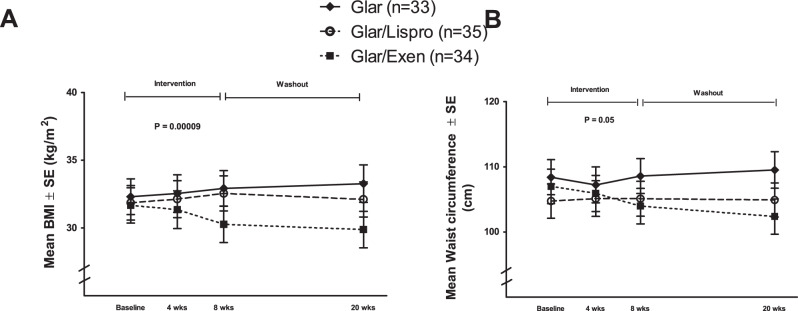
Changes over time in anthropometric variables. **A** body mass index (BMI) and **B** waist circumference. Data are presented as mean values ± standard error. Generalized estimating equation (GEE) model was used to test time-treatment interaction effect during the 8-week intervention. Two-tailed *P*-values <0.05 were considered statistically significant (multiple comparisons not considered). Number of participants: Glar *n* = 33; Glar/Lispro *n* = 35; Glar/Exen *n* = 34.

Figure 3 shows the changes in metabolic measures in the three groups over the 8-week intervention and subsequent 12-week washout. As shown in Fig. 3A, A1c changed differentially between the three groups during the intervention (time-treatment interaction *p* = 0.03), with greater decrease in the Glar/Exenatide and Glar/Lispro arms than in the Glar group. During the washout, however, A1c returned to a similar level in all three groups. With fasting glucose, each therapy yielded a similar pattern of reduction during the intervention followed by rising levels during the washout (Fig. 3B). All three therapies induced an increase in ISSI-2 by 8-weeks but this improvement in beta-cell function was lost during the washout (Fig. 3C), thereby mirroring the pattern of changes in A1c. The changes in insulin sensitivity (Matsuda index) were more variable but did not differ between the groups (Fig. 3D). In contrast, the interventions induced a clear differential effect on BMI, which was lowered only by Glar/Exenatide (time-treatment interaction *p* = 0.0001) (Fig. 4A). A similar differential effect (*p* = 0.05) was seen in the reduction of waist circumference by Glar/Exenatide (Fig. 4B).

**Fig. 5:**
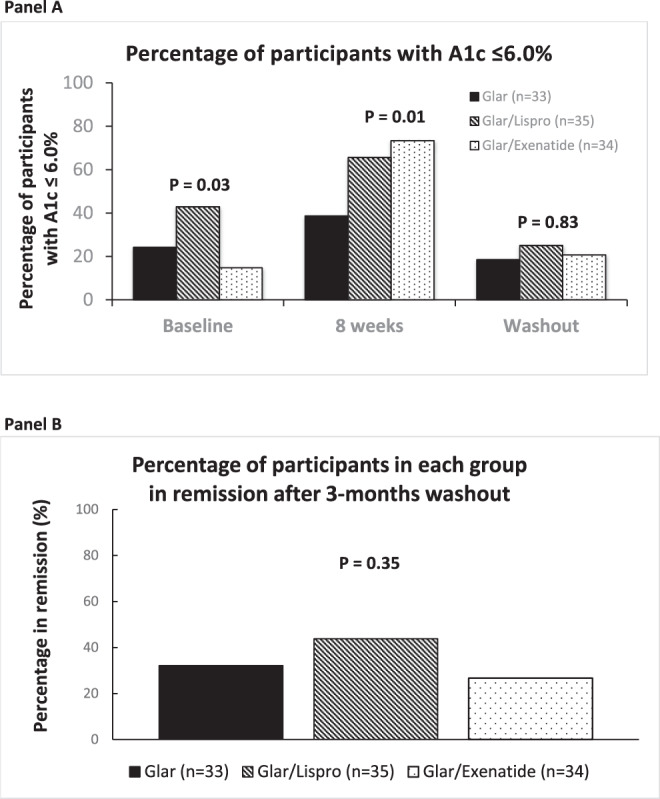
Percentages of participants with A1c ≤ 6.0% and remission of diabetes. **A** Percentage of participants in each group with A1c ≤ 6.0% at baseline, 8-weeks and washout, respectively. **B** Percentage of participants in each group with remission of diabetes after 3-months washout. Chi-square test was used to compare proportions between groups. Two-tailed *P*-values < 0.05 were considered statistically significant (multiple comparisons not considered). Number of participants: Glar *n* = 33; Glar/Lispro *n* = 35; Glar/Exen *n* = 34.

The additional categorical outcomes assessing glycemic control yielded further insight into the relative effects of the interventions both on therapy and after 3-months washout. Of note, the groups differed in the prevalence of A1c ≤ 6.0% at baseline, with this degree of glycemic control achieved on pre-study medications in 42.9% of the participants randomized to Glar/Lispro, 24.2% of those assigned to Glar, and 14.7% of those in Glar/Exenatide (*p* = 0.03) (Fig. 5A). After 8-weeks on their randomly allocated interventions, these proportions increased in all three groups but to different degrees, with the highest prevalence of A1c ≤ 6.0% now observed in Glar/Exenatide (73.3%), followed by Glar/Lispro (65.6%) and Glar (38.7%; *p* = 0.01). These differential effects of the interventions were then completely lost at washout (*p* = 0.83). Finally, the prevalence of remission of diabetes after 3-months washout did not differ significantly between these three insulin-based interventions (Glar/Exenatide 26.7%, Glar/Lispro 43.8%, Glar 32.1%; *p* = 0.35; Fig. 5B).

There were no episodes of severe hypoglycemia during the trial or differences between the groups in other adverse events (Supplementary Table 1). There were more individuals in the Glar/Lispro group with at least 1 episode of capillary glucose <4.0 mmol/l than in the Glar/Exenatide or Glar groups (*p* = 0.04), with no such differences at more stringent hypoglycemic thresholds of <3.5 mmol/l or ≤3.0 mmol/l (Table 3). There were no differences between the groups in the number of hypoglycemic events or the rate of events per patient-year at any of the glycemic thresholds (<4.0 mmol/l, <3.5 mmol/l, ≤3.0 mmol/l; Table 3).

**Table 3:** Incidence and rates of hypoglycemia in the treatment arms, with hypoglycemia assessed at three different thresholds in three ways: (i) number of individuals, (ii) number of events, and (iii) rate per patient-year

	Glargine	Glargine + Lispro	Glargine + Exenatide	*P*
(I) Number of individuals:^a^				
Blood glucose <4.0 mmol/l	15	26	23	0.04
Blood glucose <3.5 mmol/l	10	12	6	0.27
Blood glucose ≤3.0 mmol/l	1	8	5	0.06
(II) Number of events:^b^				
Blood glucose <4.0 mmol/l	68	158	130	0.10
Blood glucose <3.5 mmol/l	21	34	17	0.49
Blood glucose ≤3.0 mmol/l	3	11	5	0.19
(III) Rate of events per patient-year:^c^				
Blood glucose <4.0 mmol/l	13.7	30.6	27.6	0.14
Blood glucose <3.5 mmol/l	4.2	6.6	3.6	0.45
Blood glucose ≤3.0 mmol/l	0.6	2.1	1.1	0.21

^a^For comparison of number of individuals, Chi-Square test or Fisher exact test was used to compare proportions.

^b^For comparison of number of events, negative binomial regression was used to compare counts between the groups because the event could occur multiple times in some participants and we assumed that each participant has recurrent events according to an individual poisson event rate and that the distribution for the number of events for each participant in the total population follows negative binomial distribution.

^c^For comparison of rate per patient-year during the trial, rates were defined as total number of events divided by total weeks of follow-up to account for varying intervals of follow-up^35^. Negative binomial regression with the offset of the amount log(observed time period in years or weeks) was used to account for different lengths of follow-up of participants.

## Discussion

In this trial, we demonstrate that adding exenatide to basal insulin glargine in early T2DM did not further enhance underlying beta-cell function beyond the improvement achieved with glargine alone. Nevertheless, Glar/Exenatide yielded greater reduction in A1c and weight than either Glar or Glar/Lispro. However, none of these benefits persisted over 12-weeks washout, such that the prevalence of diabetes remission ultimately did not differ between these three insulin-based interventions. These findings suggest that the addition of exenatide to glargine in early T2DM offers beneficial on-treatment (pharmacologic) effects that do not appear to change the underlying biology determining beta-cell function or the capacity to achieve diabetes remission.

There has been limited previous investigation of basal insulin/GLP1-RA combination therapy early in the course of T2DM. In a study of 28 patients with diabetes of mean duration 8.66 years, 4-weeks treatment with the combination of lixisenatide and glargine increased glucose-stimulated insulin secretion (measured on intravenous glucose tolerance test performed 30-minutes after lixisenatide administration) to a greater extent than did 4-weeks treatment with either medication alone^19^. In patients with shorter duration of diabetes, GLP1-RA monotherapy has been shown to improve beta-cell function as compared to placebo^20, 21^ but, to our knowledge, the metabolic effects of its combination with basal insulin have not been evaluated in this setting.

Against this background, the current trial was designed to assess both on-treatment and subsequent post-therapy effects of combining exenatide and glargine in early T2DM. While the on-treatment pharmacologic effects of an intervention (such as A1c and BMI at 8-weeks) are of universal interest in all clinical studies, the post-therapy biologic impact of Glar/Exenatide (i.e. in the absence of pharmacologic effects) holds particular importance in the clinical context of the current trial. Specifically, when administering short-term insulin therapy in early T2DM, an underlying goal of the intervention is to ameliorate the reversible component of beta-cell dysfunction that exists early in the natural history of diabetes^15^. Ideally, the resultant recovery of beta-cell function may then facilitate the capacity to maintain glucose homeostasis after stopping the short-term intervention, thereby enabling the possibility of achieving diabetes remission. These considerations thus informed three distinct features of the design of this trial. First, exenatide was not administered prior to the OGTTs (and glargine was held the night before) in order to exclude/limit pharmacologic effects on the measurement of beta-cell function during the intervention and thereby gain insight into underlying endogenous function. Second, the 12-week washout was designed to enable assessment of the clinical outcome of diabetes remission. Third, Glar/Exenatide was compared with two active comparator interventions (Glar/Lispro and Glar) that have been shown to have post-therapy effects on beta-cell function and resultant capacity to induce glycemic remission^9–13^.

With this trial design, we show that all three interventions improved beta-cell function at 8-weeks but that there were no significant differences in their ability to do so. Similarly, all three interventions could induce remission 3-months later, but again with no significant differences in the proportion of participants achieving this outcome. Taken together, these data suggest that, despite its beneficial on-treatment effects on A1c and weight, the addition of exenatide to basal insulin in early T2DM did not further enhance underlying beta-cell function or the capacity to achieve diabetes remission. These findings are consistent with existing literature on the absence of post-therapy effects of GLP1-RAs in early T2DM. In the Restoring Insulin Secretion (RISE) Adult Medication Study, 3-months of metformin followed by 9-months of liraglutide yielded on-treatment reductions in A1c and weight coupled with improved beta-cell function; however, these effects did not persist after 3-months washout^22^. In the LIraglutide and Beta-cell RepAir (LIBRA) trial, the robust enhancement of beta-cell function that was sustained over 48-weeks treatment with liraglutide was completely lost within 2-weeks of stopping the therapy^21^. An earlier study by Bunck et al.^23^ yielded equivocal findings by showing that the beneficial effect of 1-year of exenatide on beta-cell function was lost upon 4-weeks washout. Although a subsequent analysis after 3-years of treatment reported a modest post-washout benefit in disposition index, this observation was drawn from only 52% of the original study population (36 individuals) such that definitive conclusions cannot be drawn^24^. Overall, current thinking holds that GLP1-RAs provide pharmacologic enhancement of insulin secretion but likely do not modify the underlying pathophysiology of beta-cell dysfunction in a way that yields sustained benefits off-therapy. The current study now extends the literature by showing that this concept similarly applies to the combined administration of exenatide and basal insulin in early T2DM (a setting in which exogenous insulin offers sustained metabolic effects that exenatide does not further enhance).

Previous studies have shown that glargine/exenatide combination therapy provides glycemic and clinical benefits (weight loss and lower risk of hypoglycemia) in patients with longstanding diabetes, even when compared to basal/bolus insulin regimens^3, 4, 25^. The current study shows that these advantageous features even extend to well-controlled patients who are early in the course of T2DM. Indeed, in this study population with median 3.5 years duration of diabetes and mean baseline A1c 6.6%, treatment with Glar/Exenatide yielded lower baseline-adjusted A1c and BMI at 8-weeks than either Glar/Lispro or Glar alone, without high rates of hypoglycemia. Moreover, almost three quarters (73.3%) of those randomized to Glar/Exenatide achieved A1c ≤ 6.0% at 8-weeks. These data suggest that, rather than short-term intervention aimed at disease modification and remission, this combination may warrant consideration as ongoing treatment in early T2DM for its glycemic and clinical benefits. Indeed, amidst current debate on the appropriate timing for initiating basal insulin/GLP1-RA combination therapy^8^, the current trial provides evidence in support of this treatment strategy even within the first few years after diagnosis and in the setting of good glycemic control. Further studies of the early initiation of basal insulin/GLP1-RA therapy are needed to evaluate the durability of the on-treatment metabolic effects observed in this trial.

A limitation of this trial is that beta-cell function was assessed with OGTT-based surrogate indices rather than direct measurement on clamp studies, which potentially might have provided greater capacity for detecting subtle differences. Conversely, the four OGTT-based indices of beta-cell function yielded fully consistent findings and are validated measures that have been widely used in previous studies^15, 21, 26–28^. The distinct nature of the three interventions in this study dictated the need for an open-label design. Similarly, these interventions dictated that a necessary limitation was differential self-monitoring of glucose in the three arms. Specifically, whereas the Glar/Lispro arm performed glucose measurements before and after all three meals and at bedtime that guided dose adjustments, it was felt that we could not reasonably make the same request of participants in the Glar/Exenatide or Glar arms since only a subset of such measurements would impact dosing in these two groups (e.g. measurements at fasting and bedtime in both arms, and before dinner in the Glar/Exenatide arm). Nevertheless, despite less frequent glucose monitoring than that of the Glar/Lispro arm, Glar/Exenatide yielded greater reduction in A1c. Another factor to consider is that the current findings may not necessarily extend to other GLP1-RA formulations that could be combined with basal insulin in early T2DM. Of note, despite the theoretical rationale supporting the choice of exenatide to complement glargine^16, 17^, a recent meta-analysis found that, when combined with basal insulin, long-acting GLP1-RAs yielded better glucose-lowering and weight control than did short-acting formulations, coupled with better gastrointestinal tolerability^7^. Also, the relative impact of a short-acting GLP1-RA may be greater in patients with good glycemic control (where A1c may be more dependent on postprandial glucose). Thus, future studies should evaluate other GLP1-RA formulations in combination with basal insulin in early T2DM. Lastly, the current study cannot provide commentary on the beta-cell impact of these three insulin-based interventions versus a non-insulin-based control; rather, this study was designed to enable comparison between these three therapies in early T2DM.

In conclusion, the addition of exenatide to basal insulin glargine in patients with T2DM of modest duration did not further enhance underlying beta-cell function beyond the improvement achieved with glargine alone. Nevertheless, at this early point in the natural history of diabetes, Glar/exenatide outperformed Glar/Lispro and Glar in yielding greater reduction in weight and A1c, with 73.3% of patients achieving A1c ≤ 6.0% at 8-weeks. However, none of these benefits persisted over 12-weeks washout, such that the prevalence of diabetes remission ultimately did not differ between the three insulin-based interventions. Thus, these data do not support the addition of exenatide to short-term basal insulin therapy as a strategy for enhancing the achievability of beta-cell functional recovery and glycemic remission in early T2DM.

## Methods

This trial was conducted at a single academic center (Leadership Sinai Centre for Diabetes, Mount Sinai Hospital, Toronto) and was approved by the Mount Sinai Hospital Research Ethics Board. The trial was registered at ClinicalTrials.Gov NCT02194595. The trial was conducted in accordance with Good Clinical Practice and the principles of the Declaration of Helsinki, and all participants provided written informed consent.

### Study population

Participants were recruited between 23/09/2014 and 20/07/2021. Inclusion criteria included age 30–80 years; T2DM of ≤7 years duration; treatment with up to 2 oral anti-diabetic medications (with no change in dose/regimen in the preceding 4 weeks); and screening A1c between 6.0–9.5% if on no anti-diabetic medications or between 5.5 and 9.0% if on anti-diabetic medication. Exclusion criteria included treatment with insulin or a GLP1-RA; renal dysfunction (estimated glomerular filtration rate <30 ml/min); history of pancreatitis; and familial or personal history of Multiple Endocrine Neoplasia type 2 or medullary thyroid carcinoma.

### Randomization and Intervention (8-weeks)

Participants were randomized to the three interventions in a 1:1:1 manner. The computer-generated random allocation sequence was prepared by the Applied Health Research Centre (Toronto), which provided participant allocation in sealed envelopes for opening at the baseline visit.

Participants were instructed to stop any oral anti-diabetic medications the day before their baseline visit. They then completed an overnight fast before undergoing a 2-h, 75 g OGTT the next morning at this visit. All participants received instruction on healthy lifestyle practices for managing T2DM and were encouraged to follow these practices for the duration of the trial. They were then randomized to one of the following three interventions for 8-weeks:

(I) Glargine (Glar) – participants in this arm were started on insulin glargine at dose 0.12 units/kg, administered once daily at bedtime. They were instructed to perform self-monitoring of capillary glucose at least twice per day at bedtime and fasting, with other measurements at their discretion. These measurements were sent to study staff and enabled the titration of the glargine dose to target fasting glucose ≤5.3 mmol/l (target in ORIGIN Trial^13^). Participants withheld their glargine dose on the night before their 4-week visit and their 8-week visit, respectively, before undergoing overnight fast for the OGTT that was performed at these visits.

(II) Glargine + Lispro (Glar/Lispro) – participants in this arm received multiple daily insulin injection therapy consisting of basal insulin glargine and thrice-daily pre-meal lispro, with starting total daily doses of 0.3 units/kg, apportioned as 60% bolus insulin (0.18 units/kg/day) and 40% basal insulin (0.12 units/kg/day). Participants performed self-monitoring at least 4 times/day (including fasting glucose every day; before each meal at least 4 times/week; 2-hours after each meal at least 4 times/week; and at bedtime at least 4 times/week). These glucose measurements enabled the titration of insulin doses to target fasting glucose ≤5.3 mmol/l and 2-hour postprandial glucose *<*8 mmol/l. On the night before the 4-week visit and the 8-week visit, the last insulin dose was lispro before dinner, with no bedtime basal insulin (followed by overnight fast for the OGTT the next morning).

(III) Glargine + Exenatide (Glar/Exenatide) – participants in this arm were started on glargine once daily at bedtime (at starting dose 0.12 units/kg) and twice-daily exenatide 5 μg sc administered before breakfast and dinner. At their 4-week visit, the doses of exenatide were increased to 10 μg sc before breakfast and dinner. Participants performed self-monitoring at fasting, before dinner and at bedtime, with glargine doses titrated to target fasting glucose ≤5.3 mmol/l. On the night before the 4-week visit and the 8-week visit, they administered exenatide before dinner, with no bedtime basal insulin (followed by overnight fast for the OGTT the next morning).

The 8-week duration of intervention in this trial was selected based on two factors. First, a previous meta-analysis has shown that the administration of short-term intensive insulin therapy for 2–4 weeks can induce remission of T2DM that persists for 1-year thereafter in 46% of participants^10^. Second, the usual titration schedule for exenatide is 4 weeks at 5ug twice a day before progressing to the standard dose of 10ug twice a day. Thus, 8-weeks treatment provided 4 weeks of therapy with the standard dose of exenatide in the Glar/Exenatide arm (i.e. representing a duration of intervention that can potentially induce remission).

### Washout (12-weeks)

After the 8-week visit, participants stopped the assigned intervention and entered the 12-week washout. During this time, they were instructed to follow healthy lifestyle practices for the management of T2DM off any anti-diabetic medications. If their fasting or pre-meal capillary glucose measurements exceeded 10.0 mmol/l on four occasions during any week, participants were instructed to notify study staff to arrange an early washout visit, followed by return to clinical care.

### Physiologic Indices on OGTT

All OGTTs at baseline, 4-weeks, 8-weeks, and 20-weeks were performed in the morning after overnight fast. Anti-diabetic medications were held on the morning of the OGTT. During each OGTT, venous blood samples were drawn for measurement of glucose, C-peptide, and insulin at fasting and at 10-, 20-, 30-, 60-, 90-, and 120-min following ingestion of the 75 g glucose load. Specific insulin was measured with the Roche Elecsys-1010 immunoassay analyzer and electrochemiluminescence immunoassay kit. C-peptide was measured with the Roche Modular system and electrochemiluminescence immunoassay kit (Roche Diagnostics).

Whole-body insulin sensitivity was measured by Matsuda index^29^ and hepatic insulin resistance was assessed by Homeostasis Model Assessment (HOMA-IR)^30^. Beta-cell function was assessed by Insulin Secretion-Sensitivity Index-2 (ISSI-2), which is a validated OGTT-derived measure of beta-cell function that is analogous to the disposition index obtained from the intravenous glucose tolerance test (ivGTT), against which it has been directly validated^31, 32^. ISSI-2 is defined as the product of (i) insulin secretion measured by the ratio of area-under-the-insulin-curve to area-under-the-glucose-curve on the OGTT and (ii) insulin sensitivity measured by Matsuda index. Additional measures of beta-cell function included (i) insulinogenic index/HOMA-IR, (ii) ΔCpep_0-120_/Δgluc_0-120_×Matsuda index and (iii) ΔISR_0-120_/Δgluc_0-120_ × Matsuda index (where ISR is the pre-hepatic insulin secretion rate determined by C-peptide deconvolution)^15^.

### Outcomes and power

The primary outcome was mean ISSI-2 over the 8-week intervention. The secondary outcomes were baseline-adjusted measures of A1c at 8-weeks, ISSI-2 at 20-weeks and A1c at 20-weeks. Additional outcomes were baseline-adjusted measures at 8-weeks and 20-weeks for supplementary beta-cell indices, insulin sensitivity/resistance, fasting and 2-hour glucose, and anthropometric measures (weight, BMI, waist). Additional secondary outcomes pertaining to vascular function (baseline-adjusted endothelial function at 8-weeks and 20-weeks) will be reported separately. Diabetes remission was defined as A1c < 6.5% after 3-months off any anti-diabetic medications, as per the recent consensus definition of remission^33^. The pre-trial power calculation indicated that a sample size of 32 patients per arm would provide 80% power to detect a minimum difference in log-scaled ISSI-2 of 0.25 (or an equivalent difference in ISSI-2 of 44) between the Glar/Exenatide and Glar arms, with a significant level (alpha) of 0.05, based on the standard deviation of log-scaled ISSI-2 (0.46) that was previously noted in patients with early T2DM receiving short-term insulin therapy and assuming a correlation of r = 0.4 between ISSI-2 measurements at baseline, 4-weeks and 8-weeks (based on a previous observational study with repeated ISSI-2 measurements)^34^.

### Statistical analyses

Statistical analyses were conducted with SAS 9.4 (SAS Institute, Cary, NC) and on an intention-to-treat basis. Two-tailed *P* values < 0.05 were considered statistically significant. Continuous variables were tested for normality of distribution, and natural log transformations of skewed variables were used where necessary. Characteristics of the study arms at baseline and intervention were compared by Analysis of Variance (normally distributed variables) or Kruskal–Wallis test (skewed variables), or either Chi-Square test or Fisher exact test (categorical variables; Table 1). Continuous outcomes at 8-weeks and 20-weeks were compared between the groups by ANCOVA with adjustment for their baseline measurements (Table 2). The primary outcome of mean ISSI-2 over the 8-week intervention was compared between the three treatment groups by generalized estimating equation (GEE) model, wherein the treatment effect and time effect were examined (Table 2). Other longitudinal changes in outcomes of interest from baseline to 8-weeks were evaluated using the same GEE model, wherein the treatment effect, time effect and time-treatment interaction were examined (Figs. 3 and 4). The proportions of participants with A1c ≤ 6.0% at baseline, 8-weeks and washout, respectively, (Fig. 5A) and with remission of diabetes after 3-months washout (Fig. 5B) were compared between the three groups.

### Reporting summary

Further information on research design is available in the Nature Research Reporting Summary linked to this article.

## Supplementary information

Supplementary Information

Reporting Summary

## Data Availability

De-identified data can be made available under restricted access from the corresponding author, for academic purposes, subject to a material transfer agreement and approval of the Mount Sinai Hospital Research Ethics Board. Individual participant data that underlie the results reported in this article can be made available by this mechanism, after de-identification, to achieve the aims in the approved proposal. The study protocol can also be made available in this way. This data access mechanism will be available beginning 9 months and ending 36 months following publication of this article. Source data are provided with this paper.

## Source data

Source Data

## Code availability

The code used for analysis can be made available under restricted access from the corresponding author upon request for academic purposes, subject to approval of the Mount Sinai Hospital Research Ethics Board.
