# Supplementary material for: The metabolic effects of adding exenatide to basal insulin therapy when targeting remission in early type 2 diabetes in a randomized clinical trial
Source: Nat Commun. 2022 Oct 16;13:6109. doi: 10.1038/s41467-022-33867-9 (PMC9573864; doi:10.1038/s41467-022-33867-9)
Supplement: Supplementary file 1 — Supplementary Information [file 41467_2022_33867_MOESM1_ESM.pdf]

Supplementary Information

Supplementary Figure 1: Glucose response on the oral glucose tolerance tests (OGTT) at (A) baseline, (B) 8-weeks, and (C) washout. Data are presented as mean values ± standard error.

Supplementary Figure 2: Insulin response on the oral glucose tolerance test (OGTT) at (A) baseline, (B) 8-weeks, and (C) washout. Data are presented as mean values ± standard error.

Supplementary Table 1: Comparison of adverse events between treatment arms (number of individuals with event)

Supplementary Figure 1: Glucose response on the OGTT at (A) baseline, (B) 8-weeks, and (C) washout Data are presented as mean values ± standard error.

Panel A: At baseline

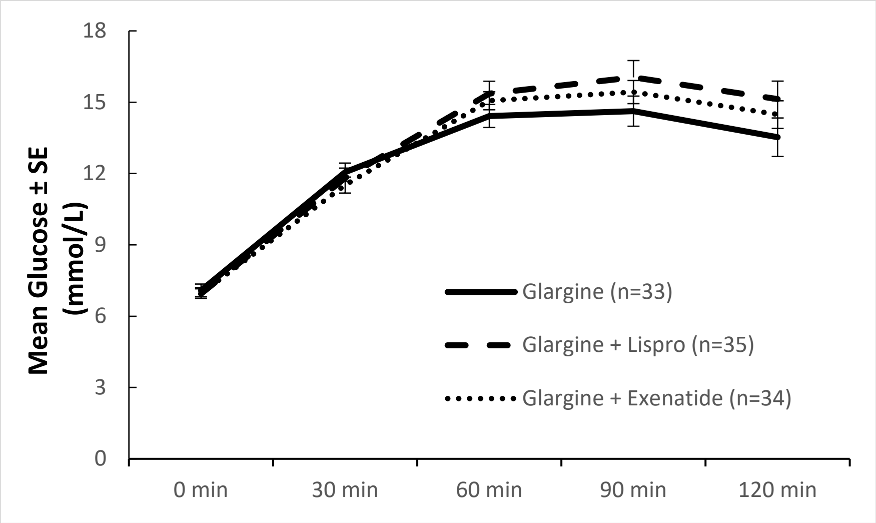

Panel B: At 8-weeks

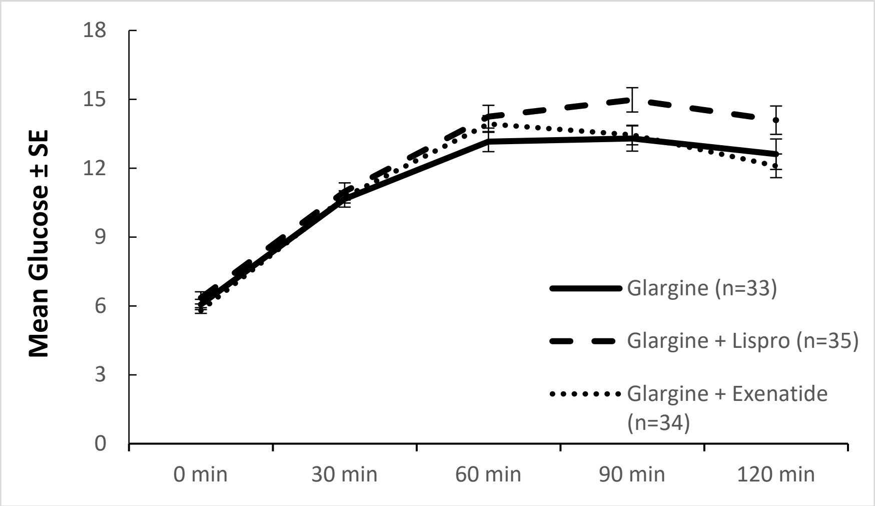

Panel C: At washout

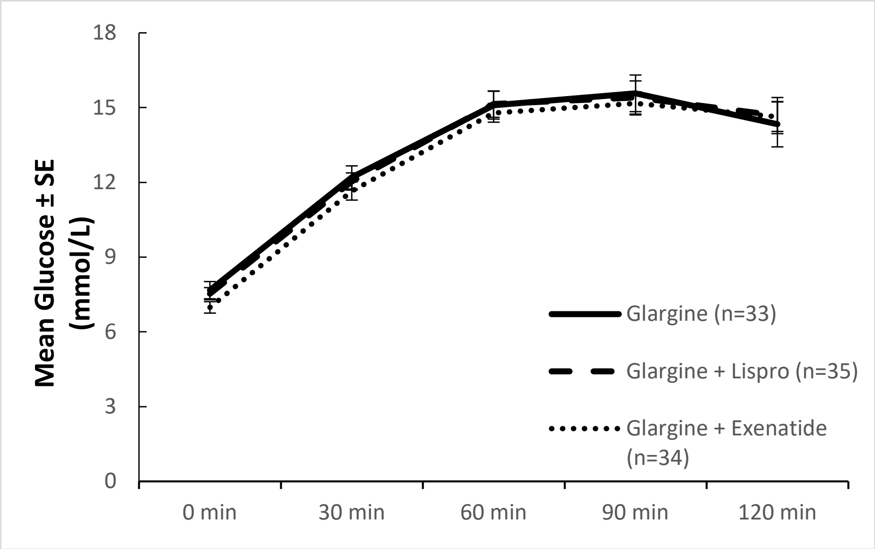

Supplementary Fig. 2: Insulin response on the OGTT at (A) baseline, (B) 8-weeks, and (C) washout
Data are presented as mean values ± standard error.

Panel A: At baseline

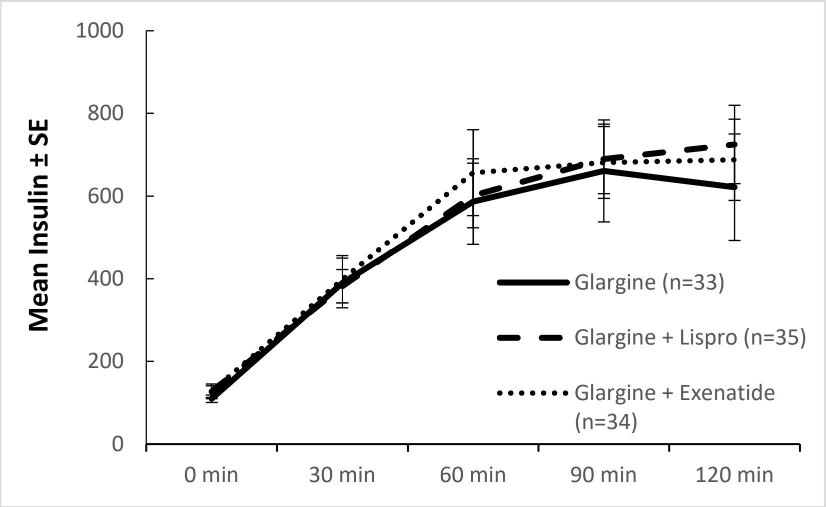

Panel B: At 8-weeks

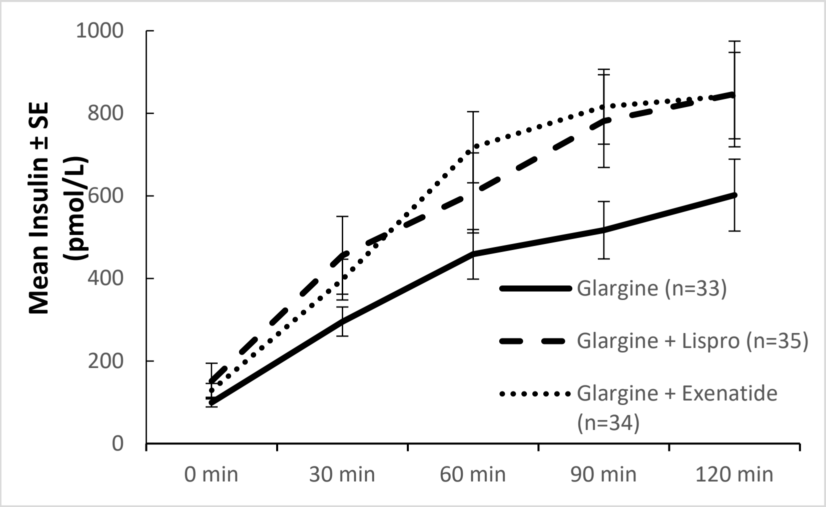

Panel C: At washout

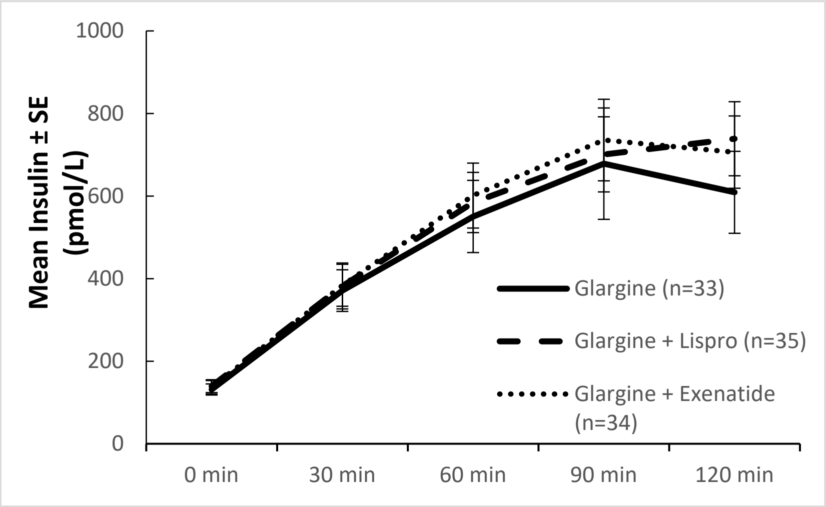

Supplementary Table 1: Comparison of adverse events between treatment arms (number of individuals with event)

	Glargine (n=33)	Glargine + Lispro (n=35)	Glargine + Exenatide (n=34)	P
**Hypoglycemia**				
Any	16	26	22	0.09
Severe	0	0	0	*
**Gastrointestinal**				
Bowel obstruction	0	1	0	
Vomitted	0	0	1	1.00
**Respiratory**				
Sinusitis	0	2	1	0.77
Upper respiratory tract infection	2	1	0	0.32
Cough	0	1	0	1.00
Flu-like illness	1	2	1	1.00
**Neurologic**				
Headache	0	0	1	0.66
**Urogenital**				
Urinary tract infection	1	2	0	0.65
**Other**				
Fatigue	0	0	1	0.66

*No severe hypoglycemia events occurred in any group, such that no test of comparison was applied.

The proportions of participants experiencing adverse events during the trial were compared between the three groups by Chi-Square test or Fisher exact test
