# Supplementary material for: The metabolic effects of adding exenatide to basal insulin therapy when targeting remission in early type 2 diabetes in a randomized clinical trial
Source: Nat Commun. 2022 Oct 16;13:6109. doi: 10.1038/s41467-022-33867-9 (PMC9573864; doi:10.1038/s41467-022-33867-9)
Supplement: Supplementary file 2 — Reporting Summary [file 41467_2022_33867_MOESM2_ESM.pdf]

nature portfolio

Corresponding author(s): Prof. Ravi Retnakaran

Last updated by author(s): Sep 7, 2022

Reporting Summary

Nature Portfolio wishes to improve the reproducibility of the work that we publish. This form provides structure for consistency and transparency in reporting. For further information on Nature Portfolio policies, see our Editorial Policies and the Editorial Policy Checklist.

Statistics

For all statistical analyses, confirm that the following items are present in the figure legend, table legend, main text, or Methods section.

n/a	Confirmed	
☐	☒	The exact sample size (*n*) for each experimental group/condition, given as a discrete number and unit of measurement
☒	☐	A statement on whether measurements were taken from distinct samples or whether the same sample was measured repeatedly
☐	☒	The statistical test(s) used AND whether they are one- or two-sided<br>*Only common tests should be described solely by name; describe more complex techniques in the Methods section.*
☐	☒	A description of all covariates tested
☐	☒	A description of any assumptions or corrections, such as tests of normality and adjustment for multiple comparisons
☐	☒	A full description of the statistical parameters including central tendency (e.g. means) or other basic estimates (e.g. regression coefficient) AND variation (e.g. standard deviation) or associated estimates of uncertainty (e.g. confidence intervals)
☐	☒	For null hypothesis testing, the test statistic (e.g. *F*, *t*, *r*) with confidence intervals, effect sizes, degrees of freedom and *P* value noted<br>*Give P values as exact values whenever suitable.*
☒	☐	For Bayesian analysis, information on the choice of priors and Markov chain Monte Carlo settings
☒	☐	For hierarchical and complex designs, identification of the appropriate level for tests and full reporting of outcomes
☐	☒	Estimates of effect sizes (e.g. Cohen's *d*, Pearson's *r*), indicating how they were calculated

Our web collection on statistics for biologists contains articles on many of the points above.

Software and code

Policy information about availability of computer code

Data collection	There was no software involved in data collection.
Data analysis	Statistical analyses were conducted with SAS 9.4 (SAS Institute, Cary, NC)

For manuscripts utilizing custom algorithms or software that are central to the research but not yet described in published literature, software must be made available to editors and reviewers. We strongly encourage code deposition in a community repository (e.g. GitHub). See the Nature Portfolio guidelines for submitting code & software for further information.

Data

Policy information about availability of data

All manuscripts must include a data availability statement. This statement should provide the following information, where applicable:

- Accession codes, unique identifiers, or web links for publicly available datasets
- A description of any restrictions on data availability
- For clinical datasets or third party data, please ensure that the statement adheres to our policy

De-identified data can be made available under restricted access from the corresponding author, for academic purposes, subject to a material transfer agreement and approval of the Mount Sinai Hospital Research Ethics Board.

Human research participants

Policy information about studies involving human research participants and Sex and Gender in Research.

Reporting on sex and gender	Self-reported data on sex were collected and post-hoc exploratory analyses were performed to determine if sex influenced the findings. It should be noted, however, that these were post-hoc analyses, recognizing that the sample size was not designed to be large enough for robust sex-based analyses. Data were not collected on gender, as a potential effect of gender role on the impact of the interventions was not hypothesized a priori.
Population characteristics	The study population consisted of 102 participants (58 male, 44 female) with age 58 ± 10 years, type 2 diabetes of median 3.5 years duration (interquartile range 1.8-5.5 years), baseline A1c 6.6% ±0.7% and BMI 31.9 ±7.5 kg/m2.
Recruitment	Participants were recruited from the practices of family physicians (either by screening charts or physician referral) and in response to advertising of the study. As in most clinical trials, the possibility of self-selection bias exists but was unlikely to impact the findings of this trial due to the design (randomized trial) and outcomes under study (physiologic measures).
Ethics oversight	This study was approved by the Mount Sinai Hospital Research Ethics Board (Toronto, Canada)

Note that full information on the approval of the study protocol must also be provided in the manuscript.

Field-specific reporting

Please select the one below that is the best fit for your research. If you are not sure, read the appropriate sections before making your selection.

☒ Life sciences ☐ Behavioural & social sciences ☐ Ecological, evolutionary & environmental sciences

For a reference copy of the document with all sections, see nature.com/documents/nr-reporting-summary-flat.pdf

Life sciences study design

All studies must disclose on these points even when the disclosure is negative.

Sample size	The pre-trial power calculation indicated that a sample size of 32 patients per arm would provide 80% power to detect a minimum difference in log-scaled ISSI-2 of 0.25 (or an equivalent difference in ISSI-2 of 44) between the Glar/Exenatide and Glar arms, with a significant level (alpha) of 0.05, based on the standard deviation of log-scaled ISSI-2 (0.46) that was previously noted in patients with early T2DM receiving short-term insulin therapy and assuming a correlation of r=0.4 between ISSI-2 measurements at baseline, 4-weeks and 8-weeks.
Data exclusions	One participant withdrew from the study after completing the trial and withdrew consent for inclusion of their data (which is noted in the Consort trial profile in Figure 2). All other participants were analyzed on an intention-to-treat basis.
Replication	Sensitivity analyses were performed three times to confirm robustness of the findings. In addition, for any serum samples that showed evidence of hemolysis, insulin measurements were repeated and the statistical analyses were replicated with this dataset (which confirmed the findings)
Randomization	Participants were randomized to the 3 interventions in a 1:1:1 manner. The computer-generated random allocation sequence was prepared by the Applied Health Research Centre (Toronto), which provided participant allocation in sealed envelopes for opening at the baseline visit.
Blinding	This was an open-label trial because masking of treatment allocation would require placebo injections in the glargine arm (since glargine is administered once daily) and in the exenatide/glargine arm (since exenatide is administered twice daily, whereas pre-meal lispro is injected three times daily). Furthermore, the two approved fixed doses of exenatide (5ug bid and 10ug bid) would preclude effective self-monitoring-based dose titration in the same manner as typically applied to lispro. For these reasons, an open-label design was applied. In this context, a protection against bias is that the primary outcome of beta-cell function is a physiologic parameter that is not readily accessible to manipulation by either participants or providers.

Reporting for specific materials, systems and methods

We require information from authors about some types of materials, experimental systems and methods used in many studies. Here, indicate whether each material, system or method listed is relevant to your study. If you are not sure if a list item applies to your research, read the appropriate section before selecting a response.

Materials & experimental systems

n/a	Involved in the study
☒	☐ Antibodies
☒	☐ Eukaryotic cell lines
☒	☐ Palaeontology and archaeology
☒	☐ Animals and other organisms
☐	☒ Clinical data
☒	☐ Dual use research of concern

Methods

n/a	Involved in the study
☒	☐ ChIP-seq
☒	☐ Flow cytometry
☒	☐ MRI-based neuroimaging

Clinical data

Policy information about clinical studies

All manuscripts should comply with the ICMJEguidelines for publication of clinical research and a completedCONSORT checklist must be included with all submissions.

Clinical trial registration	The trial was registered at ClinicalTrials.Gov NCT02194595.
Study protocol	The study protocol has been included with the submission.
Data collection	The study population was recruited between 23/09/2014 and 20/07/2021. Participants were recruited in Toronto from the practices of family physicians (either by screening charts or physician referral) and in response to advertising of the study. The study was completed in Feb 2022.
Outcomes	The pre-defined primary outcome was mean ISSI-2 over the 8-week intervention. The secondary outcomes were baseline-adjusted measures of A1c at 8-weeks, ISSI-2 at 20-weeks and A1c at 20-weeks. ISSI-2 was measured on 2-hour 75g oral glucose tolerance test (OGTT). ISSI-2 is defined as the product of (i) insulin secretion measured by the ratio of area-under-the-insulin-curve to area-under-the-glucose-curve on the OGTT and (ii) insulin sensitivity measured by Matsuda index. A1c was measured by standard clinical biochemistry.
